# Dynamic analysis of a Caputo fractional-order SEIR model with a general incidence rate

**DOI:** 10.1038/s41598-025-01400-9

**Published:** 2025-05-21

**Authors:** Shenghu Xu, Yanhui Hu

**Affiliations:** School of Mathematics and Information Science, North Minzu University, Yinchuan, 750021 Ningxia People’s Republic of China

**Keywords:** Caputo fractional order, Lyapunov function, Global stability, Ecology, Evolution, Systems biology, Mathematics and computing

## Abstract

This study develops a fractional-order SEIR model with asymptomatic infections and memory effects, introducing a generalized incidence rate to better reflect the nonlinear characteristics of transmission. The Caputo fractional derivative is used to capture memory effects and non-locality, dynamically adjusting the order to adapt to complex processes, improving accuracy and fitting. Based on Lyapunov functions, we rigorously prove that the disease-free equilibrium is globally asymptotically stable when $R_0<1$, and the endemic equilibrium is globally stable when $R_0>1$. Sensitivity analysis identifies key factors influencing disease spread and control. Numerical simulations validate the theoretical results and demonstrate the advantages of the fractional-order model in capturing epidemic dynamics, which traditional integer-order models fail to capture such dynamics. This study contributes to more accurate disease modeling and provides insights for optimizing control strategies for complex infectious diseases.

## Introduction

Infectious diseases have posed a considerable challenge throughout history^1,2^, driving ongoing research. From smallpox outbreaks in ancient Egypt^3^ to the Black Death^4^ and cholera pandemics^5^ of the 19th century, more recently SARS and COVID-19 outbreaks, each event has left a lasting impact on human society. These crises have also spurred advancements in science, technology, and public health. In 1796, Edward Jenner developed the first effective vaccine, marking the beginning of modern vaccine development and ultimately leading to global smallpox eradication in the 20th century. Similarly, Louis Pasteur developed vaccines for rabies and anthrax and pioneered research improving food safety and sanitation practices.

Infectious disease models^6,7^ have undergone several significant stages of development. British researchers John Graunt and William Farr used statistical methods to analyze disease trends in the 17th century. By the early 20th century, mathematical modeling of infectious disease became more systematic. In 1927, British mathematicians Kermack^8^ and McKendrick introduced the SIR model, laying the foundation for modern infectious disease modeling. Building on this, infectious disease models expanded and improved in the second half of the 20th century. The SEIR model^9^ was developed to account for diseases where individuals may be infected but not yet contagious. In addition to fractional-order modeling methods, other mathematical tools have also been widely applied in complex system modeling in recent years, including Bayesian games^10^, Boolean control networks^11^, fractal mappings^12^, and discrete system stability analysis^13^. These methods have been applied in various fields and may provide new perspectives for future research in epidemiological dynamics.

More recently, Wang et al.^14^ further subdivided the infected compartment into symptomatic and asymptomatic individuals, allowing for more accurate representation of diseases with asymptomatic transmission. Wang et al.^14^ proposed the SEIR model as follows:

1 $$\begin{aligned} \left\{ \begin{aligned}&\frac{\textrm{d} S}{\textrm{d} t}=\Lambda -\beta S(I_{1} +I_{2})-\mu S,\\&\frac{\textrm{d} E}{\textrm{d} t}=\beta S(I_{1}+I_{2} )-(\epsilon +\mu )E,\\&\frac{\textrm{d} I_{1}}{\textrm{d} t}=p\epsilon E-(\mu +\alpha +r_{1} )I_{1},\\&\frac{\textrm{d} I_{2}}{\textrm{d} t}=q\epsilon E-(\mu +\alpha +r_{2} )I_{2},\\&\frac{\textrm{d} R}{\textrm{d} t}=r_{1} I_{1}+ r_{2} I_{2}-\mu R,\end{aligned}\right. \end{aligned}$$

where *S* denotes susceptible individuals, *E* stands for exposed individuals, $I_1(t)$ is the number of infectious individuals, who are diagnosed and symptomatic, $I_2(t)$ is the number of the infectious individuals, who are diagnosed but asymptomatic, and *R* indicates recovered individuals. The parameter $\Lambda$ represents the recruitment rate of susceptible individuals, and $\beta$ is the transmission rate through contact. $\mu$ is the natural mortality rate, and $\epsilon$ is the diagnosis rate. The proportion of symptomatic individuals among all diagnosed cases is denoted by *p*, and *q* is the proportion of asymptomatic individuals. $\alpha$ stands for the mortality rate due to the disease. Lastly, $r_1$ and $r_2$ represent the recovery rates of symptomatic and asymptomatic individuals, respectively. It is assumed that the parameters $\Lambda$ and $\mu$ are positive, while the parameters $\beta$, $\epsilon$, *p*, *q*, $\alpha$, $r_{1}$, and $r_{2}$ are non-negative, with the condition that $p + q = 1$.

“*The asymptomatic ratio* $q = 0.3166$ *aligns with empirical COVID-19 data*^14^, *where 30–40% of infections are asymptomatic. This assumption increases* $R_0$ *by 18% compared to models ignoring* $I_2$.” For more information on the model, we refer interested readers to^14^ and the reference there in.

**Fig. 1:**
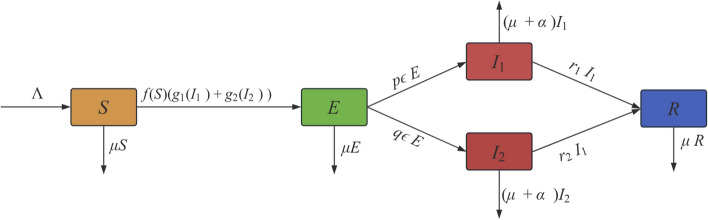
The flowchart of SEIR with general incidence rate.

Based on the model in^14^, we introduce the SEIR model with general incidence rate, The model can be described as follows, and we design a flowchart based on the general incidence rate model (Fig. 1).

2 $$\begin{aligned} \left\{ \begin{aligned}&\frac{\textrm{d} S}{\textrm{d} t}=\Lambda -f(S)(g_{1}(I_{1} )+g_{2}(I_{2} ) )-\mu S,\\ &\frac{\textrm{d} E}{\textrm{d} t}=f(S)(g_{1}(I_{1} )+g_{2}(I_{2} ) )-(\epsilon +\mu )E,\\&\frac{\textrm{d} I_{1}}{\textrm{d} t}=p\epsilon E-(\mu +\alpha +r_{1} )I_{1},\\&\frac{\textrm{d} I_{2}}{\textrm{d} t}=q\epsilon E-(\mu +\alpha +r_{2} )I_{2},\\&\frac{\textrm{d} R}{\textrm{d} t}=r_{1} I_{1}+ r_{2} I_{2}-\mu R.\end{aligned}\right. \end{aligned}$$

Fractional calculus^15–17^ expands upon traditional integer-order calculus, concentrating on the investigation of derivatives and integrals of arbitrary real or complex orders. Although the concept of fractional derivatives was first introduced by Leibniz in 1695, the field did not advance rapidly at that time. Nonetheless, in recent years, fractional calculus has emerged as a highly active research area^18–21^ propelled by its growing application to practical problems, particularly in fields such as nonlinear dynamics, control theory, signal processing, and quantum mechanics. Compared to traditional calculus, fractional calculus enhances flexibility and precision in describing complex physical phenomena and system behaviors^22–25,31^. Researchers have studied Lyapunov functions for fractional-order systems and derived key fractional-order inequalities, which play a crucial role in proving the global stability of various fractional order models^26,27^. They have also examined the incorporation of a fractional order SEIR model without asymptomatic infective individuals. The Caputo fractional derivative effectively describes the long-term dynamics of disease, such as incubation periods and immune decay. Compared to other types of fractional derivatives, the Caputo derivative is more applicable to epidemiological modeling, particularly due to its compatibility with traditional integer-order differential equations. Memory effects arise from the non-local nature of fractional derivatives, where past states influence current dynamics through a power-law kernel $(t - u)^{-\gamma }$. Therefore, we propose integrating the fractional Caputo derivative into the SEIR model with general incidence rate and asymptomatic infective individuals

3 $$\begin{aligned} \left\{ \begin{aligned}&_{0}^{C} D_{t}^{\gamma } S(t)=\Lambda -f(S)(g_{1}(I_{1} )+g_{2}(I_{2} ) )-\mu S,\\&_{0}^{C} D_{t}^{\gamma } E(t)=f(S)(g_{1}(I_{1} )+g_{2}(I_{2} ) )-(\epsilon +\mu )E,\\&_{0}^{C} D_{t}^{\gamma }I_{1} (t)=p\epsilon E-(\mu +\alpha +r_{1} )I_{1},\\&_{0}^{C} D_{t}^{\gamma }I_{2} (t)=q\epsilon E-(\mu +\alpha +r_{2} )I_{2},\\&_{0}^{C} D_{t}^{\gamma }R (t)=r_{1} I_{1}+ r_{2} I_{2}-\mu R,\end{aligned}\right. \end{aligned}$$

where $\gamma \in (0,1]$ and initial conditions

4 $$\begin{aligned} S(0)>0,E(0)>0,I_{1}(0)>0,I_{2} (0)>0,R(0)>0. \end{aligned}$$

Moreover, we assume that the functional response function *f*(*x*) and $g_{i}(x)$ satisfies the following conditions:

(A_1_): $$f(0)=0,f'(x)>0,$$ for all $$x\ge 0$$.

(A_2_): $$g_{i}(0)=0,g_{i}'(x)>0$$; $$\frac{g_{i}(x)}{x}\text {is non-increasing}$$ for all $$x\ge 0,i=1,2.$$

Condition *A*_1_ ensures $f(S)$ increases with susceptible density, while condition *A*_2_ captures the saturation effects via through the non-increasing term $g_i(I_i)/I_i$. This form not only includes the classic bilinear infection rate ( $\beta SI,\beta >0$), but also incorporates saturation effects($\frac{\beta S I}{1 + \alpha I}, \beta , \alpha > 0.$), Crowley-Martin incidence rate ( $\frac{\beta SI}{(1 + aS)(1 + bI)}, \beta , a, b > 0.$), sublinear incidence rate($\beta SI^p, \beta > 0, \ 0< p < 1.$), and generalized saturation ($\frac{\beta S \cdot I}{1 + \alpha I^\gamma }, \beta , \alpha > 0, \ \gamma \ge 1.$).

The generalized incidence rate function more accurately describes the changes in transmission caused by factors such as population size, behavioral changes, and asymptomatic individuals. In particular, asymptomatic individuals play an important role in transmission, which traditional linear models fail to capture effectively. By adjusting the transmission rates, the model can effectively simulate the effects of medical interventions and social distancing measures. By numerically simulating the adjustments of parameters $\beta _1$ and $\beta _2$, the model better adapts to different epidemic scenarios. For example, reducing the contact frequency of highly infectious groups can significantly reduce $R_0$, thereby effectively controlling the spread of the epidemic. The function enhances the model’s adaptability to various transmission mechanisms, better fits real epidemic data, and provides strong support for public health decision-making, helping to formulate more precise control strategies.

Model (3) generalizes many special cases available in the literature^26,27^. The system (3) includes the following SEIR models:

- Model of Yang et al.^26^ with$I_2 = 0$.
- Model of Almeida et al.^27^ with $f(s) = s$, $g_1(I_1) = I_1$, and $I_2 = 0$

The references in^28–30^ all study fractional-order SEIR models, which discuss the transmission processes of COVID-19, Ebola and H1N1/09 influenza. The above models are all SEIR models with bilinear infection rates. However, the model (3) with general incidence rate, brings a new difficulty if the nonlinear incidence rate combines with the effect of fractional-order derivative. *Recent studies [42,43] demonstrate Caputo models’ efficacy in HIV-malaria co-infection dynamics, supporting our frameworks versatility.*

The organization of this paper is as follows: section “Preliminaries” provides the definitions and basic results of Caputo fractional calculus. Section “Well-posedness” conducts a qualitative analysis of the model. Section “Stability analysis” focuses on the dynamical analysis of the model. In section “Sensitivity analysis”, we select common incidence functions for sensitivity analysis and provide control and prevention recommendations based on the model’s results. In section “Numerical simulations”, we present numerical simulations to verify the correctness of the theoretical findings and explore the memory and hereditary properties of the fractional-order model.

## Preliminaries

In this section, some definitions and lemmas are provided.

### Definition 2.1

^32^. For any integrable function $f:\mathbb {R}_{+} \rightarrow \mathbb {R}$, the Riemann-Liouville fractional integral is defined as

$$\begin{aligned} I_{t}^{\gamma } f(t)=\frac{1}{\Gamma (\gamma )} \int _{t_{0}}^{t} (t-u)^{\gamma -1} f(u)\textrm{d}u, \end{aligned}$$

where $$t_{0}\le t$$, $$0< \gamma <1$$.

### Definition 2.2

^32^. The Caputo fractional derivative operator of the function *f* is defined as

$$\begin{aligned} _{t_{0}}^{C} D_{t}^{\gamma } f(t)=\frac{1}{\Gamma (1-\gamma )} \int _{t_{0}}^{t} (t-u)^{-\gamma }\frac{\textrm{d} }{\textrm{d} u}f(u)\textrm{d}u, \end{aligned}$$

where $$0<\gamma <1.$$

### Definition 2.3

^32^. The two-parameters Mittag-Leffler function is defined as

$$\begin{aligned} E_{\eta _{1},\eta _{2}} (z)=\sum _{k=0}^{\infty } \frac{z^{k} }{\Gamma (k\eta _{1}+\eta _{2})},\eta _{1},\eta _{2}>0,z\in \mathbb {C}. \end{aligned}$$

In brief, let $E_{\eta _{1}} (z): = E_{\eta _{1},1} (z).$

The Laplace transform of the Caputo fractional derivative is given by

$$\begin{aligned} \mathcal {L}\{_{0}^{C} D_{t}^{\gamma }g(t) \}=s^{\gamma } F(s)-\sum _{k=0}^{n-1} s^{\gamma -k-1}{g}^{(k)}(0),n<\gamma <n-1. \end{aligned}$$

The Laplace transform of the function $t^{\eta _{2}-1} E_{\eta _{1},\eta _{2}}(\pm \lambda t^{\eta _{1}} )$ can be expressed as

$$\begin{aligned} \mathcal {L}[t^{\eta _{2}-1}E_{\eta _{1},\eta _{2}}(\pm \lambda t^{\eta _{1}} ) ]=\frac{s^{\eta _{1}-\eta _{2}} }{s^{\eta _{1} } \mp \lambda }, \end{aligned}$$

where $$F(s)=\mathcal {L}(g(t))$$.

### Lemma 2.1

^33^. Consider the following fractional-order system

$$\begin{aligned} _{t_{0}}^{C} D_{t}^{\gamma } g(t)=f(t,g(t)), \end{aligned}$$

with the initial condition $g(t_{0})=g_{t_{0}}$ and $0<\gamma <1$. The condition for the local asymptotic stability of the equilibrium points of this system is that for every eigenvalue $\eta _{i}$ of the Jacobian matrix $\frac{\partial f(t,y)}{\partial g}$, evaluated at the equilibrium points, it holds that $\left| \textrm{arg} (\eta _{i}) \right| >\frac{\gamma \pi }{2}$.

### Lemma 2.2

^22^. Let $\psi (t)\in C[0,\infty )$ satisfy $_{t_{0}}^{C} D_{t}^{\gamma } \psi (t)+b_{1}\psi (t)\le b_{2}$, $\psi (0)=\psi _{0}$, where $0<\gamma \le 1$, and $b_{1},b_{2}\in \mathbb {R}$, with $b_{1} \ne 0$. Then,

$$\begin{aligned} \psi (t)\le (\psi _{0}-\frac{b_{2}}{b_{1}} )E_{\gamma ,1}(-b_{1}t^{\gamma } ) +\frac{b_{2}}{b_{1}}, \end{aligned}$$

where $E_{\gamma ,1}(\cdot )$ is a one parameter Mittag-Leffler function.

### Lemma 2.3

^24^. Consider *u*(*t*) be a real positive differentiable function. Then, for any $t\ge t_{0}$, $0<\gamma \le 1$, and $u^{*} >0$, it follows that

$$\begin{aligned} _{t_{0} }^{C} D_{t}^{\gamma } \Psi (u(t))\le \left( 1-\frac{g(u^{*} )}{g(u(t))} \right) \; _{t_{0} }^{C} D_{t}^{\gamma } u(t), \end{aligned}$$

where $\Psi (u)=\int _{u^{*} }^{u} \frac{g(s)-g(u^{*} )}{g(s)} ds$, the function *g* is a non-negative, differentiable, and strictly increasing on $\mathbb {R}^{+}$. Note that $\Psi$ is positive on $\mathbb {R}^{+}\backslash {u^{*} }$ with $\Psi (u^{*} )=0$. In fact, $\Psi$ is differentiable and

$$\begin{aligned} \frac{d\Psi }{du} =1-\frac{g(u^{*} )}{g(u)}. \end{aligned}$$

Since $g$ is a strictly increasing function, $\Psi$ is strictly decreasing for $u < u^{*}$ and strictly increasing for $u > u^{*}$, where $u^{*}$ represents the global minimum.

## Well-posedness

### Theorem 3.1

The system (3) with initial condition (4) has a unique solution.

$$\begin{aligned} \Omega =\left\{ (S,E,I_{1},I_{2},R)\in \mathbb {R}_{+}^{5}:S,E,I_{1},I_{2},R>0,S+E+I_{1}+I_{2}+R\le S_{0}\right\} \end{aligned}$$

is positively invariant, where $S_{0}=\frac{\Lambda }{\mu }$.

### Proof

Define

$$\begin{aligned} X(t)=\begin{pmatrix}S(t) \\ E(t) \\ I_{1}(t)\\ I_{2}(t)\\ R(t)\end{pmatrix}, \quad X_{0} =\begin{pmatrix}S(0) \\ E(0) \\ I_{1}(0)\\ I_{2}(0)\\ R(0)\end{pmatrix}, \end{aligned}$$

and

$$\begin{aligned} h(X(t))=\begin{pmatrix}h_{1} (X(t)) \\ h_{2} (X(t)) \\ h_{3} (X(t))\\ h_{4} (X(t))\\ h_{5} (X(t))\end{pmatrix}=\begin{pmatrix}\Lambda -f(S)(g_{1}(I_{1} )+g_{2}(I_{2} ) )-\mu S \\ f(S)(g_{1}(I_{1} )+g_{2}(I_{2} ) )-(\epsilon +\mu )E\\ p\epsilon E-(\mu +\alpha +r_{1} )I_{1} \\ q\epsilon E-(\mu +\alpha +r_{2} )I_{2}\\ r_{1} I_{1}+ r_{2} I_{2}-\mu R\end{pmatrix}. \end{aligned}$$

The system with initial conditions can be expressed as follows

$$\begin{aligned} \left\{ \begin{matrix}_{0}^{C} D_{t}^{\gamma } X(t)=h(X(t)), \\ X(0)=X_{0}.\end{matrix}\right. \end{aligned}$$

Given conditions (*A*_1_) and (*A*_2_), we know that the Jacobian matrix $\frac{\partial h}{\partial x} = \frac{\partial (h_1, h_2, h_3, h_4, h_5)}{\partial (S, E, I_1, I_2, R)}$ is continuous on $\mathbb {R}_+^5.$ Using Remark 1.2.1 from^34^, it follows that $h$ is locally Lipschitz continuous on $\mathbb {R}_+^5$. Moreover, applying Remark 3.8 from^35^, we conclude that the system (3) with (4) has a unique solution.

To show the positivity of the solutions, we derive the following from system (3),

$$\begin{aligned}_{0 }^{C} D_{t}^{\gamma } S(t)\big | _{S=0}&=\Lambda \ge 0,\\_{0}^{C} D_{t}^{\gamma } E(t)\big | _{E=0}&=f(S)(g_{1}(I_{1} )+g_{2}(I_{2} ) )\ge 0,\\_{0}^{C} D_{t}^{\gamma } I_{1}(t)\big | _{I_{1}=0}&=p\epsilon E\ge 0,\\_{0}^{C} D_{t}^{\gamma } I_{2}(t)\big | _{I_{2}=0}&=q\epsilon E\ge 0,\\_{0}^{C} D_{t}^{\gamma }R(t)\big |_{R=0}&=r_{1} I_{1}+ r_{2} I_{2}\ge 0.\end{aligned}$$

According to the Generalized Mean Value Theorem in reference^36^, the solutions are non-negative and remain within $\mathbb {R}_{+}^{5}$.

Define $N(t)=S(t)+E(t)+I_{1}(t)+I_{2}(t)+R(t).$ From system (3), we obtain

$$\begin{aligned} _{0}^{C} D_{t}^{\gamma } N(t)&= \Lambda - \mu (S(t) + E(t) + I_{1}(t) + I_{2}(t) + R(t)) - \alpha I_{1} - \alpha I_{2} \\&\le \Lambda - \mu N(t). \end{aligned}$$

Therefore, by Lemma 2.2, we obtain the following result,

$$\begin{aligned} N(t)\le (N(0)-\frac{\Lambda }{\mu } )E_{\gamma } (-\mu t^{\gamma } )+\frac{\Lambda }{\mu }. \end{aligned}$$

By considering the asymptotic behavior of the Mittag-Leffler function as $t \rightarrow \infty$, we finally obtain $N(t)\le S_{0}$.

Thus, the region $$\Omega$$ is positively invariant, this is sufficient to analyze the dynamics of system (3) in region $$\Omega.$$$$\square$$

## Stability analysis

Firstly, we use the the next-generation matrix method^37^ to calculate $R_{0}$ of system (3), $x=(E,I_{1},I_{2},S,R)^T, x_{0} =(0,0,0,S_{0},0)^T$, where $S_{0}=\frac{\Lambda }{\mu }$, $_{0}^{C} D_{t}^{\gamma } x(t)=\mathscr {F}-\mathscr {V}$ , where

$$\begin{aligned} \mathscr {F}=\begin{pmatrix}f(S)(g_{1}(I_{1})+g_{2}(I_{2}))\\ 0\\ 0\\ 0\\ 0\end{pmatrix}, \quad \mathscr {V}=\begin{pmatrix}(\epsilon +\mu ) E\\ -p\epsilon E+(\mu +\alpha +r_{1} )I_{1} \\ -q\epsilon E+(\mu +\alpha +r_{2} )I_{2}\\ -\Lambda + f(S)(g_{1}(I_{1})+g_{2}(I_{2}))+\mu S\\ -r_{1}I_{1} -r_{2}I_{2} +\mu R \end{pmatrix}. \end{aligned}$$

Calculate the Jacobian matrix

$$\begin{aligned} F \mid _{x_{0}}= & \left. \begin{pmatrix} 0 & f(S) g_{1}'(I_{1}) & f(S) g_{2}'(I_{2}) \\ 0 & 0 & 0 \\ 0 & 0 & 0 \end{pmatrix} \right| _{x_{0}} = \begin{pmatrix} 0 & f(S_{0}) g_{1}'(0) & f(S_{0}) g_{2}'(0) \\ 0 & 0 & 0 \\ 0 & 0 & 0 \end{pmatrix}.\\ V\mid _{x_{0} }= & \begin{pmatrix}\epsilon +\mu & 0 & 0\\ -p\epsilon & \mu +\alpha +r_{1} & 0\\ -q\epsilon & 0 & \mu +\alpha +r_{2}\end{pmatrix},\quad V^{-1} =\begin{pmatrix}\frac{1}{\epsilon +\mu } & 0 & 0\\ \frac{p\epsilon }{(\epsilon +\mu )(\mu +\alpha +r_{1} )} & \frac{1}{\mu +\alpha +r_{1} } & 0\\ \frac{q\epsilon }{(\epsilon +\mu )(\mu +\alpha +r_{2})} & 0 & \frac{1}{\mu +\alpha +r_{2} } \end{pmatrix}. \end{aligned}$$

Therefore

$$\begin{aligned} R_{0} =\rho (FV^{-1} )=\frac{f(S_{0} ){g_{1}}'(0)p\epsilon }{(\epsilon +\mu )(\mu +\alpha +r_{1} )} + \frac{f(S_{0} ){g_{2}}'(0)q\epsilon }{(\epsilon +\mu )(\mu +\alpha +r_{2})}. \end{aligned}$$

Let the basic reproduction number for symptomatic individuals be $R_1 = \frac{ \Lambda \beta _1 p \epsilon }{\mu (\epsilon + \mu )(\mu + \alpha + r_1)},$ and for asymptomatic individuals be $R_2 = \frac{ \Lambda \beta _2 q \epsilon }{\mu (\epsilon + \mu )(\mu + \alpha + r_2)}.$ Then, the overall basic reproduction number is $R_0 = R_1 + R_2$, which accounts for the transmission contributions from both symptomatic and asymptomatic individuals.

The disease-free equilibrium of System (3) is given by $P_0(S_0, 0, 0, 0, 0)$, where $S_0 = \frac{\Lambda }{\mu }$. We now proceed to establish the existence and uniqueness of the positive equilibrium point, setting

5 $$\begin{aligned}\left\{ \begin{aligned}&\Lambda -f(S)(g_{1}(I_{1} )+g_{2}(I_{2} ) )-\mu S=0,\\&f(S)(g_{1}(I_{1} )+g_{2}(I_{2} ) )-(\epsilon +\mu )E=0,\\&p\epsilon E-(\mu +\alpha +r_{1} )I_{1}=0,\\&q\epsilon E-(\mu +\alpha +r_{2} )I_{2}=0,\\&r_{1} I_{1}+ r_{2} I_{2}-\mu R=0.\end{aligned}\right. \end{aligned}$$

By adding the first two equations of (5), we obtain

6 $$\begin{aligned} S = \frac{\Lambda }{\mu } - \frac{\epsilon + \mu }{\mu } E. \end{aligned}$$

From the third equation of (5), we deduce

7 $$\begin{aligned} I_{1}=\frac{p\epsilon }{\mu +\alpha +r_{1} }E. \end{aligned}$$

From the firth equation, it follows that

8 $$\begin{aligned} I_{2}=\frac{q\epsilon }{\mu +\alpha +r_{2} }E. \end{aligned}$$

Substituting them into the second equation of (5), we obtain

$$\begin{aligned} f\left( \frac{\Lambda }{\mu } - \frac{\epsilon + \mu }{\mu }E \right) \left[ g_{1} \left( \frac{p \epsilon }{\mu + \alpha + r_{1} } E \right) + g_{2} \left( \frac{q \epsilon }{\mu + \alpha + r_{2} } E \right) \right] - (\epsilon + \mu ) E =0. \end{aligned}$$

Define

$$\begin{aligned} \Psi (E)=f\left( \frac{\Lambda }{\mu } - \frac{\epsilon + \mu }{\mu }E \right) \left[ g_{1} \left( \frac{p \epsilon }{\mu + \alpha + r_{1} } E \right) + g_{2} \left( \frac{q \epsilon }{\mu + \alpha + r_{2} } E \right) \right] - (\epsilon + \mu ) E. \end{aligned}$$

It is known that $\Psi (0) = 0$ and $\Psi \left( \frac{\Lambda }{\epsilon + \mu }\right) = -\Lambda < 0$. Hence, we will study the existence and uniqueness of the positive equilibrium point within the interval $(0, \frac{\Lambda }{\epsilon + \mu })$. Taking the derivative of both sides of the equation, we get

$$\begin{aligned}{\Psi }' (E)&= -\frac{\epsilon + \mu }{\mu } {f}'\left( \frac{\Lambda }{\mu } - \frac{\epsilon + \mu }{\mu } E \right) \left[ g_{1} \left( \frac{p \epsilon }{\mu + \alpha + r_{1} } E \right) + g_{2} \left( \frac{q \epsilon }{\mu + \alpha + r_{2} } E \right) \right] \\&\quad + f \left( \frac{\Lambda }{\mu } - \frac{\epsilon + \mu }{\mu } E \right) \left[ \frac{p \epsilon }{\mu + \alpha + r_{1} } {g_{1} }' \left( \frac{p \epsilon }{\mu + \alpha + r_{1} } E \right) \right. \\&\qquad \left. + \frac{q \epsilon }{\mu + \alpha + r_{2} } {g_{2}}' \left( \frac{q \epsilon }{\mu + \alpha + r_{2} } E \right) \right] - (\epsilon + \mu ).\\ {\Psi }' (0)&= -\frac{\epsilon + \mu }{\mu } {f}'\left( \frac{\Lambda }{\mu } \right) \Big ( g_{1}(0) + g_{2}(0) \Big ) + f\left( \frac{\Lambda }{\mu } \right) \left[ \frac{p \epsilon }{\mu + \alpha + r_{1} } {g_{1} }'(0) \right. \\&\qquad \left. + \frac{q \epsilon }{\mu + \alpha + r_{2} } {g_{2}}'(0) \right] - (\epsilon + \mu ) \\&= -(\epsilon + \mu ) + f\left( \frac{\Lambda }{\mu } \right) \left[ \frac{p \epsilon }{\mu + \alpha + r_{1} } {g_{1} }'(0) + \frac{q \epsilon }{\mu + \alpha + r_{2} } {g_{2}}'(0) \right] \\&= (\epsilon + \mu ) \left[ \frac{f\left( \frac{\Lambda }{\mu } \right) p \epsilon }{(\epsilon + \mu )(\mu + \alpha + r_{1}) } {g_{1} }'(0) + \frac{f\left( \frac{\Lambda }{\mu } \right) q \epsilon }{(\epsilon + \mu )(\mu + \alpha + r_{2}) } {g_{2}}'(0) - 1 \right] \\&= (\epsilon + \mu )(R_{0} - 1). \end{aligned}$$

When $R_0 > 1$, it follows that ${\Psi }'(0) > 0$. Let $P^{*} ( S^*, E^*, I_1^*, I_2^*, R^*)$ represent an equilibrium point within the interval $(0, \frac{\Lambda }{\epsilon + \mu })$. These variables satisfy equations (6), (7), and (8). Thus, we have

$$\begin{aligned} {\Psi }' (E^{*})&= -\frac{\epsilon + \mu }{\mu } {f}'\left( \frac{\Lambda }{\mu } - \frac{\epsilon + \mu }{\mu } E^{*} \right) \left[ g_{1} \left( \frac{p \epsilon }{\mu + \alpha + r_{1} } E^{*} \right) + g_{2} \left( \frac{q \epsilon }{\mu + \alpha + r_{2} } E^{*} \right) \right] \\&\quad + f \left( \frac{\Lambda }{\mu } - \frac{\epsilon + \mu }{\mu } E^{*} \right) \left[ \frac{p \epsilon }{\mu + \alpha + r_{1} } {g_{1} }' \left( \frac{p \epsilon }{\mu + \alpha + r_{1} } E^{*} \right) \right. \\&\qquad \left. + \frac{q \epsilon }{\mu + \alpha + r_{2} } {g_{2}}' \left( \frac{q \epsilon }{\mu + \alpha + r_{2} } E^{*} \right) \right] - (\epsilon + \mu ) \\&= -\frac{\epsilon + \mu }{\mu } {f}'(S^{*}) \left( g_{1}(I_{1} ^{*}) + g_{2}(I_{2} ^{*}) \right) + f(S^{*}) \left[ \frac{p \epsilon }{\mu + \alpha + r_{1} } {g_{1} }'(I_{1} ^{*}) \right. \\&\qquad \left. +\frac{q \epsilon }{\mu + \alpha + r_{2} } {g_{2}}'(I_{2} ^{*}) \right] - (\epsilon + \mu ). \end{aligned}$$

Under the condition that $\frac{g_{1}(I_{1})}{I_{1}}$ and $\frac{g_{2}(I_{2})}{I_{2}}$ are non-increasing, it follows that for $I_{1} > 0$ and $I_{2} > 0$, we have ${g_{1}}'(I_{1}) \le \frac{g_{1}(I_{1})}{I_{1}}$ and ${g_{2}}'(I_{2}) \le \frac{g_{2}(I_{2})}{I_{2}}$, then

$$\begin{aligned} {\Psi }' (E^{*})&\le -(\epsilon + \mu ) - \frac{\epsilon + \mu }{\mu }{f}' (S^{*}) (g_{1}(I_{1}^{*}) + g_{2}(I_{2}^{*})) \\&\quad + f(S^{*}) \left( \frac{p \epsilon }{\mu + \alpha + r_{1}} \frac{g_{1} (I_{1}^{*})}{I_{1}^{*}} + \frac{q \epsilon }{\mu + \alpha + r_{2}} \frac{g_{2} (I_{2}^{*})}{I_{2}^{*}} \right) \\&= -(\epsilon + \mu ) - \frac{\epsilon + \mu }{\mu }{f}' (S^{*}) (g_{1}(I_{1}^{*}) + g_{2}(I_{2}^{*}))+ \frac{f(S^{*})}{E^{*}} (g_{1} (I_{1}^{*}) + g_{2}(I_{2}^{*})) \\&= -\frac{\epsilon + \mu }{\mu } {f}' (S^{*}) (g_{1}(I_{1}^{*}) + g_{2}(I_{2}^{*})) \\&< 0. \end{aligned}$$

Thus, when the basic reproduction number $R_{0} > 1$, there exists a unique positive equilibrium point.

### Theorem 4.1

When $R_0 < 1$, then the $P_{0}$ is global asymptotic stability.

### Proof

Define the Lyapunov function as follows

$$\begin{aligned} V_{1} (t)=C_{1}\int _{S_{0} }^{S(t) } \frac{f(\tau )-f(S_{0} )}{f(\tau )} d\tau +C_{2} E+C_{3}I_{1}+C_{4}I_{2}, \end{aligned}$$

where $C_{1}$, $C_{2}$, $C_{3}$ , $C_{4}$ are positive constant. By Lemma 2.3, it can be concluded that

$$\begin{aligned} _{0}^{C} D_{t}^{\gamma } V_{1}(t)&\le C_{1} \left( 1-\frac{f(S_{0})}{f(S)}\right) \; _{0}^{C} D_{t}^{\gamma } S(t) + C_{2}\; _{0}^{C} D_{t}^{\gamma } E(t) + C_{3}\; _{0}^{C} D_{t}^{\gamma } I_{1}(t) + C_{4}\; _{0}^{C} D_{t}^{\gamma } I_{2}(t) \\&= C_{1} \left( 1 - \frac{f(S_{0})}{f(S)} \right) \left( \Lambda - f(S)(g_{1}(I_{1}) + g_{2}(I_{2})) - \mu S - (\Lambda - \mu S_{0})\right) \\&\quad + C_{2} \left( f(S)(g_{1}(I_{1}) + g_{2}(I_{2})) - (\epsilon + \mu )E\right) \\&\quad + C_{3} \left( p \epsilon E - (\mu + \alpha + r_{1}) I_{1}\right) + C_{4} \left( q \epsilon E - (\mu + \alpha + r_{2}) I_{2}\right) \\&= C_{1} \left( 1 - \frac{f(S_{0})}{f(S)} \right) (-\mu (S - S_{0}) - f(S)(g_{1}(I_{1}) + g_{2}(I_{2}))) \\&\quad + C_{2} f(S)(g_{1}(I_{1}) + g_{2}(I_{2})) - C_{2} (\epsilon + \mu ) E \\&\quad + C_{3} p \epsilon E - C_{3} (\mu + \alpha + r_{1}) I_{1} + C_{4} q \epsilon E - C_{4} (\mu + \alpha + r_{2}) I_{2} \\&= C_{1} (-\mu (S - S_{0})) \left( 1 - \frac{f(S_{0})}{f(S)} \right) - C_{1} f(S)(g_{1}(I_{1}) + g_{2}(I_{2})) \\&\quad + C_{1} f(S_{0})(g_{1}(I_{1}) + g_{2}(I_{2})) + C_{2} f(S)(g_{1}(I_{1}) + g_{2}(I_{2}))- C_{2} (\epsilon + \mu ) E \\&\quad + C_{3} p \epsilon E - C_{3} (\mu + \alpha + r_{1}) I_{1} + C_{4} q \epsilon E - C_{4} (\mu + \alpha + r_{2}) I_{2}, \end{aligned}$$

Let $C_{1} = C_{2} = 1$, we have

$$\begin{aligned} _{0}^{C} D_{t}^{\gamma } V_{1}(t)&\le -\mu (S - S_{0}) \left( 1 - \frac{f(S_{0})}{f(S)}\right) + f(S_{0})(g_{1}(I_{1}) + g_{2}(I_{2}))- (\epsilon + \mu ) E \\&\quad + C_{3} p \epsilon E - C_{3} (\mu + \alpha + r_{1}) I_{1} + C_{4} q \epsilon E - C_{4} (\mu + \alpha + r_{2}) I_{2} \\&= -\mu (S - S_{0}) \left( 1 - \frac{f(S_{0})}{f(S)}\right) - (\epsilon + \mu ) E + C_{3} p \epsilon E + C_{4} q \epsilon E \\&\quad + (f(S_{0}) g_{1}(I_{1}) - C_{3} (\mu + \alpha + r_{1}) I_{1}) + (f(S_{0}) g_{2}(I_{2}) - C_{4} (\mu + \alpha + r_{2}) I_{2}). \end{aligned}$$

If $C_{3}\ge \frac{f(S_{0})}{(\mu + \alpha + r_{1})}\frac{g_{1}(I_{1})}{I_{1}}$ and $C_{4}\ge \frac{f(S_{0})}{(\mu + \alpha + r_{2})}\frac{g_{2}(I_{2})}{I_{2}}$, then

$$\begin{aligned} f(S_{0}) g_{1}(I_{1}) - C_{3} (\mu + \alpha + r_{1}) I_{1}\le 0,\quad f(S_{0}) g_{2}(I_{2}) - C_{4} (\mu + \alpha + r_{2}) I_{2}\le 0. \end{aligned}$$

Under the condition that $\frac{g_{1}(I_{1})}{I_{1}}$ and $\frac{g_{2}(I_{2})}{I_{2}}$ are non-increasing, it follows that

$$\begin{aligned} \frac{g_{1}(I_{1})}{I_{1}}\le \lim _{x \rightarrow 0^{+}} \frac{g_{1}(I_{1})}{I_{1}},\quad \frac{g_{2}(I_{2})}{I_{2}}\le \lim _{x \rightarrow 0^{+}} \frac{g_{2}(I_{2})}{I_{2}}. \end{aligned}$$

To determine a specific value for $C_{3}$ and $C_{4}$, we can consider the limit as $I_{1}$,$I_{2}$ approaches $0^{+}$. Taking the limit, we have

$$\begin{aligned} \lim _{I_{1} \rightarrow 0^{+}} \frac{g_{1}(I_{1})}{I_{1}}=\lim _{I_{1} \rightarrow 0^{+}} \frac{g_{1}(I_{1}) - g_{1}(0)}{I_{1} - 0} = {g_{1}}'(0), \quad \lim _{I_{2} \rightarrow 0^{+}} \frac{g_{2}(I_{2})}{I_{2} } =\lim _{I_{2} \rightarrow 0^{+}} \frac{g_{2}(I_{2}) - g_{2}(0)}{I_{2} - 0} = {g_{2}}'(0), \end{aligned}$$

then let

$$\begin{aligned} C_{3} = \frac{f(S_{0})}{\mu + \alpha + r_{1}} {g_{1}}'(0),\quad C_{4} = \frac{f(S_{0})}{\mu + \alpha + r_{2}} {g_{2}}'(0), \end{aligned}$$

then

$$\begin{aligned} _{0}^{C} D_{t}^{\gamma } V_{1}(t)&\le -\mu (S - S_{0})\left( 1 - \frac{f(S_{0})}{f(S)}\right) - (\epsilon + \mu ) E + \frac{f(S_{0}) {g_{1}}'(0)}{\mu + \alpha + r_{1}} p \epsilon E + \frac{f(S_{0}) {g_{2}}'(0)}{\mu + \alpha + r_{2}} q \epsilon E \\&= -\mu (S - S_{0})\left( 1 - \frac{f(S_{0})}{f(S)}\right) + (\epsilon + \mu )\left( \frac{f(S_{0}) {g_{1}}'(0)}{(\epsilon + \mu )(\mu + \alpha + r_{1})} p \epsilon \right. \\&\quad + \left. \frac{f(S_{0}) {g_{2}}'(0)}{(\epsilon + \mu )(\mu + \alpha + r_{2})} q \epsilon - 1\right) E \\&= -\mu (S - S_{0})\left( 1 - \frac{f(S_{0})}{f(S)}\right) + (\epsilon + \mu ) (R_{0} - 1) E.\end{aligned}$$

According to the mean value theorem, it follows that $-\mu (S - S_{0})\left( 1 - \frac{f(S_{0})}{f(S)}\right) \le 0$, thus, if $R_{0} \le 1$, then $_{0}^{C} D_{t}^{\gamma } V_{1}(t)\le 0$.

Moreover, $$_{0}^{C} D_{t}^{\gamma } V_{1}(t)= 0$$ if and only if $$P=P_{0}$$. Therefore, according to LaSalle’s invariance principle, when $$R_{0} < 1$$, the disease-free equilibrium point $$P_{0}$$ is globally asymptotically stable in the interior of $$\Omega.$$$$\square$$

### Theorem 4.2

When $R_0 > 1$, then the $P^{*}$ is global asymptotic stability.

### Proof

Let the Lyapunov function be defined as follows

$$\begin{aligned} V_{2} (t)=C_{1}\int _{S^{*} }^{S} \frac{f(\tau )-f(S^{*} )}{f(\tau )} d\tau +C_{2} \int _{E^{*} }^{E} \frac{\tau -E^{*} }{\tau }d\tau +C_{3}\int _{I_{1} ^{*} }^{I_{1} }\frac{\tau -I_{1}^{*} }{\tau } d\tau +C_{4}\int _{I_{2} ^{*} }^{I_{2}}\frac{\tau -I_{2}^{*} }{\tau } d\tau , \end{aligned}$$

where $C_{1}$, $C_{2}$, $C_{3}$, $C_{4}$ are positive constant. By Lemma 2.3, it follows that

$$\begin{aligned} _{0}^{C} D_{t}^{\gamma } V_{2}(t)&\le C_{1} \left( 1 - \frac{f(S^{*})}{f(S)} \right) \;_{0}^{C} D_{t}^{\gamma } S(t) + C_{2} \left( 1 - \frac{E^{*}}{E} \right) \; _{0}^{C} D_{t}^{\gamma } E(t)\\&\quad + C_{3} \left( 1 - \frac{I_{1}^{*}}{I_{1}} \right) \; _{0}^{C} D_{t}^{\gamma } I_{1}(t) + C_{4} \left( 1 - \frac{I_{2}^{*}}{I_{2}} \right) \;_{0}^{C} D_{t}^{\gamma } I_{2}(t). \end{aligned}$$

Thus, we can get

$$\begin{aligned} _{0}^{C} D_{t}^{\gamma } V_{2}(t)&\le C_{1} \left( 1 - \frac{f(S^{*})}{f(S)} \right) \left[ \Lambda - f(S)(g_{1}(I_{1}) + g_{2}(I_{2})) - \mu S \right. \\&\qquad - \left. \left( \Lambda - f(S^{*})(g_{1}(I_{1}^{*}) + g_{2}(I_{2}^{*})) - \mu S^{*} \right) \right] \\&\quad + C_{2} \left( 1 - \frac{E^{*}}{E} \right) \left[ f(S)(g_{1}(I_{1}) + g_{2}(I_{2})) - (\epsilon + \mu )E \right. \\&\qquad - \left. \left( f(S^{*})(g_{1}(I_{1}^{*}) + g_{2}(I_{2}^{*})) - (\epsilon + \mu )E^{*} \right) \right] \\&\quad + C_{3} \left( 1 - \frac{I_{1}^{*}}{I_{1}} \right) \left[ p \epsilon E - (\mu + \alpha + r_{1}) I_{1} - \left( p \epsilon E^{*} - (\mu + \alpha + r_{1}) I_{1}^{*} \right) \right] \\&\quad + C_{4} \left( 1 - \frac{I_{2}^{*}}{I_{2}} \right) \left[ q \epsilon E - (\mu + \alpha + r_{2}) I_{2} - \left( q \epsilon E^{*} - (\mu + \alpha + r_{2}) I_{2}^{*} \right) \right] \\&= C_{1} \left( 1 - \frac{f(S^{*})}{f(S)} \right) \left[ -\mu (S - S^{*}) - \left( f(S)g_{1}(I_{1}) - f(S^{*})g_{1}(I_{1}^{*}) \right) \right. \\&\qquad - \left. \left( f(S)g_{2}(I_{2}) - f(S^{*})g_{2}(I_{2}^{*}) \right) \right] \\&\quad + C_{2} \left( 1 - \frac{E^{*}}{E} \right) \left[ \left( f(S)g_{1}(I_{1}) - f(S^{*})g_{1}(I_{1}^{*}) \right) + \left( f(S)g_{2}(I_{2}) - f(S^{*})g_{2}(I_{2}^{*}) \right) \right. \\&\qquad - \left. (\epsilon + \mu )(E - E^{*})\right] \\&\quad + C_{3} \left( 1 - \frac{I_{1}^{*}}{I_{1}} \right) \left[ p \epsilon (E - E^{*}) - (\mu + \alpha + r_{1})(I_{1} - I_{1}^{*})\right] \\&\quad + C_{4} \left( 1 - \frac{I_{2}^{*}}{I_{2}} \right) \left[ q \epsilon (E - E^{*}) - (\mu + \alpha + r_{2})(I_{2} - I_{2}^{*})\right] \\&= C_{1} \left( 1 - \frac{f(S^{*})}{f(S)} \right) \left[ -\mu (S - S^{*}) - f(S^{*})g_{1}(I_{1}^{*})\left( \frac{f(S)g_{1}(I_{1})}{f(S^{*})g_{1}(I_{1}^{*})} - 1 \right) \right. \\&\qquad - \left. f(S^{*})g_{2}(I_{2}^{*})\left( \frac{f(S)g_{2}(I_{2})}{f(S^{*})g_{2}(I_{2}^{*})} - 1 \right) \right] \\&\quad + C_{2} \left( 1 - \frac{E^{*}}{E} \right) \left[ f(S^{*})g_{1}(I_{1}^{*})\left( \frac{f(S)g_{1}(I_{1})}{f(S^{*})g_{1}(I_{1}^{*})} - 1 \right) \right. \\&\qquad + \left. f(S^{*})g_{2}(I_{2}^{*})\left( \frac{f(S)g_{2}(I_{2})}{f(S^{*})g_{2}(I_{2}^{*})} - 1 \right) \right] \\&\quad - C_{2} \left( 1 - \frac{E^{*}}{E} \right) (\epsilon + \mu )E^{*}\left( \frac{E}{E^{*}} - 1 \right) \\&\quad + C_{3} \left( 1 - \frac{I_{1}^{*}}{I_{1}} \right) \left[ p \epsilon E^{*} \left( \frac{E}{E^{*}} - 1 \right) - (\mu + \alpha + r_{1}) I_{1}^{*}\left( \frac{I_{1}}{I_{1}^{*}} - 1 \right) \right] \\&\quad + C_{4} \left( 1 - \frac{I_{2}^{*}}{I_{2}} \right) \left[ q \epsilon E^{*} \left( \frac{E}{E^{*}} - 1 \right) - (\mu + \alpha + r_{2}) I_{2}^{*}\left( \frac{I_{2}}{I_{2}^{*}} - 1 \right) \right] . \end{aligned}$$

Due to $p\epsilon E^{*} =(\mu +\alpha +r_{1} )I_{1}^{*}$ and $q\epsilon E^{*} =(\mu +\alpha +r_{2} )I_{2}^{*}$, we can get

$$\begin{aligned} _{0}^{C} D_{t}^{\gamma } V_{2}(t)&\le C_{1}\left[ -\mu (S - S^{*}) \left( 1 - \frac{f(S^{*})}{f(S)}\right) \right] \\&\quad - C_{1} f(S^{*}) g_{1}(I_{1}^{*}) \left( \frac{f(S) g_{1}(I_{1})}{f(S^{*}) g_{1}(I_{1}^{*})} - 1\right) \left( 1 - \frac{f(S^{*})}{f(S)}\right) \\&\quad - C_{1} f(S^{*}) g_{2}(I_{2}^{*}) \left( \frac{f(S) g_{2}(I_{2})}{f(S^{*}) g_{2}(I_{2}^{*})} - 1\right) \left( 1 - \frac{f(S^{*})}{f(S)}\right) \\&\quad + C_{2} f(S^{*}) g_{1}(I_{1}^{*}) \left( \frac{f(S) g_{1}(I_{1})}{f(S^{*}) g_{1}(I_{1}^{*})} - 1\right) \left( 1 - \frac{E^{*}}{E}\right) \\&\quad + C_{2} f(S^{*}) g_{2}(I_{2}^{*}) \left( \frac{f(S) g_{2}(I_{2})}{f(S^{*}) g_{2}(I_{2}^{*})} - 1\right) \left( 1 - \frac{E^{*}}{E}\right) \\&\quad -C_{2}(\epsilon + \mu )E^{*} \left( 1 - \frac{E^{*}}{E} \right) \left( \frac{E}{E^{*}} - 1 \right) \\&\quad + C_{3} p \epsilon E^{*} \left( \frac{E}{E^{*}} - 1\right) \left( 1 - \frac{I_{1}^{*}}{I_{1}}\right) - C_{3} (\mu + \alpha + r_{1}) I_{1}^{*} \left( \frac{I_{1}}{I_{1}^{*}} - 1\right) \left( 1 - \frac{I_{1}^{*}}{I_{1}}\right) \\&\quad + C_{4} q \epsilon E^{*} \left( \frac{E}{E^{*}} - 1\right) \left( 1 - \frac{I_{2}^{*}}{I_{2}}\right) - C_{4} (\mu + \alpha + r_{2}) I_{2}^{*} \left( \frac{I_{2}}{I_{2}^{*}} - 1\right) \left( 1 - \frac{I_{2}^{*}}{I_{2}}\right) \\&= C_{1}\left[ -\mu (S - S^{*}) \left( 1 - \frac{f(S^{*})}{f(S)}\right) \right] \\&\quad - C_{1} f(S^{*}) g_{1}(I_{1}^{*}) \left( \frac{f(S)g_{1}(I_{1})}{f(S^{*}) g_{1}(I_{1}^{*})} - \frac{g_{1}(I_{1})}{g_{1}(I_{1}^{*})} - 1 + \frac{f(S^{*})}{f(S)}\right) \\&\quad - C_{1} f(S^{*}) g_{2}(I_{2}^{*}) \left( \frac{f(S)g_{2}(I_{2})}{f(S^{*}) g_{2}(I_{2}^{*})} - \frac{g_{2}(I_{2})}{g_{2}(I_{2}^{*})} - 1 + \frac{f(S^{*})}{f(S)}\right) \\&\quad + C_{2} f(S^{*}) g_{1}(I_{1}^{*}) \left( \frac{f(S)g_{1}(I_{1})}{f(S^{*}) g_{1}(I_{1}^{*})} - \frac{f(S)g_{1}(I_{1}) E^{*}}{f(S^{*}) g_{1}(I_{1}^{*}) E} - 1 + \frac{E^{*}}{E}\right) \\&\quad + C_{2} f(S^{*}) g_{2}(I_{2}^{*}) \left( \frac{f(S)g_{2}(I_{2})}{f(S^{*}) g_{2}(I_{2}^{*})} - \frac{f(S)g_{2}(I_{2}) E^{*}}{f(S^{*}) g_{2}(I_{2}^{*}) E} - 1 + \frac{E^{*}}{E}\right) \\&\quad - C_{2} (\epsilon + \mu ) E^{*} \left( \frac{E}{E^{*}} - 2 + \frac{E^{*}}{E}\right) \\&\quad + C_{3} p \epsilon E^{*} \left( \frac{E}{E^{*}} - \frac{E I_{1}^{*}}{E^{*} I_{1}} - 1 + \frac{I_{1}^{*}}{I_{1}} - \frac{I_{1}}{I_{1}^{*}} + 2 - \frac{I_{1}^{*}}{I_{1}}\right) \\&\quad + C_{4} q \epsilon E^{*} \left( \frac{E}{E^{*}} - \frac{E I_{2}^{*}}{E^{*} I_{2}} - 1 + \frac{I_{2}^{*}}{I_{2}} - \frac{I_{2}}{I_{2}^{*}} + 2 - \frac{I_{2}^{*}}{I_{2}}\right) . \end{aligned}$$

We choose $C_{1} = C_{2} = 1$ , as $f(S^{*} )(g_{1}(I_{1}^{*} )+g_{2}(I_{2}^{*} ) )=(\epsilon +\mu )E^{*}$, then

$$\begin{aligned} _{0}^{C} D_{t}^{\gamma } V_{2}(t)\le&-\mu (S - S^{*}) \left( 1 - \frac{f(S^{*})}{f(S)}\right) \\&- f(S^{*}) g_{1}(I_{1}^{*}) \left( \frac{f(S) g_{1}(I_{1})}{f(S^{*}) g_{1}(I_{1}^{*})} - \frac{g_{1}(I_{1})}{g_{1}(I_{1}^{*})} - 1 + \frac{f(S^{*})}{f(S)}\right. \\&\quad \left. - \frac{f(S) g_{1}(I_{1})}{f(S^{*}) g_{1}(I_{1}^{*})} + \frac{f(S) g_{1}(I_{1}) E^{*}}{f(S^{*}) g_{1}(I_{1}^{*}) E} + 1 - \frac{E^{*}}{E} + \frac{E}{E^{*}} - 2 + \frac{E^{*}}{E}\right) \\&- f(S^{*}) g_{2}(I_{2}^{*}) \left( \frac{f(S) g_{2}(I_{2})}{f(S^{*}) g_{2}(I_{2}^{*})} - \frac{g_{2}(I_{2})}{g_{2}(I_{2}^{*})} - 1 + \frac{f(S^{*})}{f(S)}\right. \\&\quad \left. - \frac{f(S) g_{2}(I_{2})}{f(S^{*}) g_{2}(I_{2}^{*})} + \frac{f(S) g_{2}(I_{2}) E^{*}}{f(S^{*}) g_{2}(I_{2}^{*}) E} + 1 - \frac{E^{*}}{E} + \frac{E}{E^{*}} - 2 + \frac{E^{*}}{E}\right) \\&+ C_{3} p \epsilon E^{*} \left( \frac{E}{E^{*}} - \frac{E I_{1}^{*}}{E^{*} I_{1}} + 1 - \frac{I_{1}}{I_{1}^{*}}\right) \\&+ C_{4} q \epsilon E^{*} \left( \frac{E}{E^{*}} - \frac{E I_{2}^{*}}{E^{*} I_{2}} + 1 - \frac{I_{2}}{I_{2}^{*}}\right). \end{aligned}$$

Let $C_{3}=\frac{f(S^{*} ) g_{1}(I_{1}^{*} )}{p\epsilon E^{*} } ,C_{4} =\frac{f(S^{*} ) g_{2}(I_{2}^{*} )}{q\epsilon E^{*} }$, we have

$$\begin{aligned} _{0}^{C} D_{t}^{\gamma } V_{2}(t) \le&-\mu \left( S - S^{*}\right) \left( 1 - \frac{f(S^{*})}{f(S)} \right) \\&- f(S^{*}) g_{1} (I_{1}^{*}) \left( -\frac{g_{1}(I_{1})}{g_{1}(I_{1}^{*})} + \frac{I_{1} }{I_{1} ^{*} }+ \frac{f(S^{*})}{f(S)} + \frac{f(S)g_{1}(I_{1})E^{*}}{f(S^{*})g_{1}(I_{1}^{*})E} + \frac{E I_{1}^{*}}{E^{*} I_{1}} - 3 \right) \\&- f(S^{*}) g_{2} (I_{2}^{*}) \left( -\frac{g_{2}(I_{2})}{g_{2}(I_{2}^{*})} +\frac{I_{2} }{I_{2} ^{*} }+ \frac{f(S^{*})}{f(S)} + \frac{f(S)g_{2}(I_{2})E^{*}}{f(S^{*})g_{2}(I_{2}^{*})E} + \frac{E I_{2}^{*}}{E^{*} I_{2}} - 3 \right). \end{aligned}$$

Let

$$\begin{aligned} L_{1}= & -f(S^{*}) g_{1} (I_{1}^{*}) \left( -\frac{g_{1}(I_{1})}{g_{1}(I_{1}^{*})} + \frac{I_{1}}{I_{1}^{*}} + \frac{f(S^{*})}{f(S)} + \frac{f(S) g_{1}(I_{1}) E^{*}}{f(S^{*}) g_{1}(I_{1}^{*}) E} + \frac{E I_{1}^{*}}{E^{*} I_{1}} - 3 \right) ,\\ L_{2}= & -f(S^{*}) g_{2} (I_{2}^{*}) \left( -\frac{g_{2}(I_{2})}{g_{2}(I_{2}^{*})} + \frac{I_{2}}{I_{2}^{*}} + \frac{f(S^{*})}{f(S)} + \frac{f(S) g_{2}(I_{2}) E^{*}}{f(S^{*}) g_{2}(I_{2}^{*}) E} + \frac{E I_{2}^{*}}{E^{*} I_{2}} - 3 \right) . \end{aligned}$$

Considering $$H(x) = x - 1 - \ln x$$, then

$$\begin{aligned} L_{1}&= -f(S^{*}) g_{1} (I_{1}^{*}) \left[ -H\left( \frac{g_{1}(I_{1})}{g_{1}(I_{1}^{*})}\right) + H\left( \frac{I_{1}}{I_{1}^{*}}\right) + H\left( \frac{f(S^{*})}{f(S)}\right) \right. \\&\quad + \left. H\left( \frac{f(S) g_{1}(I_{1}) E^{*}}{f(S^{*}) g_{1}(I_{1}^{*}) E}\right) + H\left( \frac{E I_{1}^{*}}{E^{*} I_{1}}\right) \right]. \end{aligned}$$

Let

$$\begin{aligned} F(I_{1}) = H\left( \frac{g_{1}(I_{1})}{g_{1}(I_{1}^{*})}\right) - H\left( \frac{I_{1}}{I_{1}^{*}}\right) . \end{aligned}$$

If $F(I_{1}) \le 0$, then $L_{1} \le 0$, thus we need to prove that $F(I_{1}) \le 0$,

$$\begin{aligned} {F}' (I_{1})&= \left( 1-\frac{g_{1}(I_{1} ^{*})}{g_{1}(I_{1} )}\right) \frac{{g_{1}}'(I_{1} )}{g_{1}(I_{1} ^{*})} - \left( 1-\frac{I_{1} ^{*}}{I_{1} }\right) \frac{1}{I_{1} ^{*}}\\&= \frac{g_{1}(I_{1} )-g_{1}(I_{1} ^{*})}{g_{1}(I_{1} )g_{1}(I_{1} ^{*})} {g_{1}}'(I_{1} ) - \frac{I_{1} -I_{1} ^{*}}{I_{1} I_{1} ^{*}}. \end{aligned}$$

Since $G_{1} (I_{1} ) = \frac{g(I_{1} )}{I_{1} }$ is non-increasing, we can get when $I_{1}>0$, ${g_{1} }'(I_{1} ) \le \frac{g(I_{1} )}{I_{1} }$, then

$$\begin{aligned} {F}'(I_{1} ) \le \frac{1}{I_{1} } \left( \frac{g_{1}(I_{1} )}{g_{1}(I_{1} ^{*})} - \frac{I_{1} }{I_{1} ^{*}}\right) . \end{aligned}$$

**In case 1**, when $I_{1} \ge I_{1} ^{*}$, we have $\frac{g_{1}(I_{1} )}{I_{1} } \le \frac{g_{1}(I_{1} ^{*})}{I_{1} ^{*}}$, which implies $\frac{g_{1}(I_{1} )}{g_{1}(I_{1} ^{*})} \le \frac{I_{1} }{I_{1} ^{*}}$, Therefore, ${F}'(I) \le 0$ , for $F(I^{*}) = 0$, it follows that $F(I_{1} ) \le 0.$

**In case 2**, when $I _{1} \le I_{1} ^{*}$, we have $\frac{g_{1}(I_{1} )}{I_{1} } \ge \frac{g_{1}(I_{1} ^{*})}{I_{1} ^{*}}$, which leads to $\frac{g_{1}(I_{1} )}{g_{1}(I_{1} ^{*})} \ge \frac{I_{1} }{I_{1} ^{*}}$, thus ${F}'(I_{1} ) \ge 0$ and since $F(I_{1} ^{*}) = 0$, we can conclude that $F(I_{1} ) \le 0.$

In conclusion, for all values of $I_1$, we have $F(I_1) \le 0$, which leads to $L_1 \le 0$. By applying a similar argument, we can also deduce that $L_2 \le 0$. Also, by the mean value theorem, we can deduce that $-\mu (S-S^{*})\left( 1-\frac{f(S^{*})}{f(S)}\right) \le 0$. Thus, we can conclude that

$$\begin{aligned} _{0}^{C} D_{t}^{\gamma } V_{2}(t) = -\mu (S-S^{*})\left( 1-\frac{f(S^{*})}{f(S)}\right) + L_{1} + L_{2} \le 0. \end{aligned}$$

Therefore, if $$R_{0} > 1$$, then $$_{0}^{C} D_{t}^{\gamma } V_{2}(t)\le 0$$, and $$_{0}^{C} D_{t}^{\gamma } V_{2}(t)=0$$ if and only if $$P=P^{*}$$, according to LaSalle’s invariance principle, this implies that the positive equilibrium point $$P^*$$ is asymptotically stable within the interior of $$\Omega.$$$$\square$$

## Sensitivity analysis

To verify the correctness of the theoretical results, we selected a commonly incidence rate function for sensitivity analysis and numerical simulation, we consider $f(S)=S$, $g_{1}(I)=\beta _{1} I_{1}$ and $g_{2}(I)=\beta _{2} I_{2}$, we have

9 $$\begin{aligned} \left\{ \begin{aligned}&_{0}^{C} D_{t}^{\gamma } S(t)=\Lambda -S(\beta _{1} I_{1} +\beta _{2} I_{2})-\mu S,\\ &_{0}^{C} D_{t}^{\gamma } E(t)= S(\beta _{1} I_{1}+\beta _{2} I_{2} )-(\epsilon +\mu )E,\\&_{0}^{C} D_{t} I_{1}(t)=p \epsilon E-(\mu +\alpha +r_{1} )I_{1},\\&_{0}^{C} D_{t} I_{2}(t)=q \epsilon E-(\mu +\alpha +r_{2} )I_{2},\\&_{0}^{C} D_{t}^{\gamma } R(t)=r_{1} I_{1}+ r_{2}^{\gamma } I_{2}-\mu R. \end{aligned}\right. \end{aligned}$$

System (9) always has a disease-free equilibrium $P_{0} (S_{0},0,0,0,0)$, where $S_{0}=\frac{\Lambda }{\mu }$, the basic reproduction number for system (9) is

$$\begin{aligned} R_{0} =\frac{ \Lambda \beta _{1} p \epsilon }{\mu (\epsilon +\mu )(\mu +\alpha +r_{1} )} + \frac{ \Lambda \beta _{2} q \epsilon }{\mu (\epsilon +\mu )(\mu +\alpha +r_{2} )}, \end{aligned}$$

and $$R_{1}=\frac{ \Lambda \beta _{1} p \epsilon }{\mu (\epsilon +\mu )(\mu +\alpha +r_{1} )}, R_{2}=\frac{ \Lambda \beta _{2} q \epsilon }{\mu (\epsilon +\mu )(\mu +\alpha +r_{2} )}$$, $$R_{0}=R_{1}+R_{2}.$$

We calculate the partial derivatives of the $R_{0}$ with respect to the relevant parameters as follows,

$$\begin{aligned} \frac{\partial R_{0}}{\partial \alpha }= & -\left( \frac{R_{1}}{\mu +\alpha +r_{1} }+\frac{R_{2}}{\mu +\alpha +r_{2} }\right) ,\\ \frac{\partial R_{0}}{\partial r_{1} }= & -\frac{R_{1}}{\mu +\alpha +r_{1} },\quad \frac{\partial R_{0}}{\partial r_{2} } =-\frac{R_{2}}{\mu +\alpha +r_{2} },\\ \frac{\partial R_{0}}{\partial \Lambda }= & \frac{\beta _{1}p\epsilon }{\mu (\epsilon +\mu )(\mu +\alpha +r_{1})} +\frac{\beta _{2}q\epsilon }{\mu (\epsilon +\mu )(\mu +\alpha +r_{2})},\\ \frac{\partial R_{0}}{\partial \beta _{1}}= & \frac{\Lambda p\epsilon }{\mu (\epsilon +\mu )(\mu +\alpha +r_{1})}, \quad \frac{\partial R_{0}}{\partial \beta _{2}} =\frac{\Lambda q\epsilon }{\mu (\epsilon +\mu )(\mu +\alpha +r_{2})},\\ \frac{\partial R_{0}}{\partial p}= & \frac{\Lambda \beta _{1}\epsilon }{\mu (\epsilon +\mu )(\mu +\alpha +r_{1})}, \quad \frac{\partial R_{0}}{\partial q} =\frac{\Lambda \beta _{2}\epsilon }{\mu (\epsilon +\mu )(\mu +\alpha +r_{2})},\\ \frac{\partial R_{0}}{\partial \epsilon }= & \frac{\mu }{\epsilon +\mu } \frac{\Lambda \beta _{1}p }{\mu (\epsilon +\mu )(\mu +\alpha +r_{1})}+\frac{\mu }{\epsilon +\mu } \frac{\Lambda \beta _{2}q}{\mu (\epsilon +\mu )(\mu +\alpha +r_{2})},\\ \frac{\partial R_{0}}{\partial \mu }= & -\left( \frac{R_{1}}{\mu }+\frac{R_{1}}{\epsilon +\mu }+\frac{R_{1}}{\mu +\alpha +r_{1} }+\frac{R_{2}}{\mu }+\frac{R_{2}}{\epsilon +\mu }+\frac{R_{2}}{\mu +\alpha +r_{2} }\right) . \end{aligned}$$

It can be observed that the partial derivatives of the basic reproduction number with respect to parameters $\Lambda$, $\beta _{1}$, $\beta _{2}$, $p$, $q$, and $\epsilon$ are positive, indicating that the basic reproduction number increases as these parameters increase. On the other hand, the partial derivatives with respect to parameters $\mu$, $\alpha$, $r_{1}$, and $r_{2}$ are negative, indicating that the basic reproduction number decreases as these parameters decrease.

According to reference^38^, the normalized forward sensitivity index of a parameter $\rho$ is defined as

$$\begin{aligned} \varphi _{\rho }^{R_{0}}=\frac{\partial R_{0}}{\partial \rho } \frac{\rho }{\left| R_{0} \right| }. \end{aligned}$$

Therefore, the influence of these parameters on $R_0$ is given by the sensitivity indices as follows

$$\begin{aligned} \varphi _{\alpha }^{R_{0}}= & \frac{\partial R_{0}}{\partial \alpha }\cdot \frac{\alpha }{R_{0}}=-\left( \frac{\alpha }{\mu +\alpha +r_{1}}\cdot \frac{R_{1}}{R_{0}} +\frac{\alpha }{\mu +\alpha +r_{2}}\cdot \frac{R_{2}}{R_{0}}\right) ,\\ \varphi _{\Lambda }^{R_{0}}= & \frac{\partial R_{0}}{\partial \Lambda }\cdot \frac{\Lambda }{R_{0}}=1,\quad \varphi _{\beta _{1} }^{R_{0}}=\frac{\partial R_{0}}{\partial \beta _{1}}\cdot \frac{\beta _{1}}{R_{0}}=\frac{R_{1}}{R_{0}},\quad \varphi _{\beta _{2} }^{R_{0}}=\frac{\partial R_{0}}{\partial \beta _{2}}\cdot \frac{\beta _{2}}{R_{0}}=\frac{R_{2}}{R_{0}},\\ \varphi _{r_{1}}^{R_{0}}= & \frac{\partial R_{0}}{\partial r_{1}}\cdot \frac{r_{1} }{R_{0}}=-\frac{r_{1}}{\mu +\alpha +r_{1}}\cdot \frac{R_{1}}{R_{0}},\quad \varphi _{r_{2}}^{R_{0}}=\frac{\partial R_{0}}{\partial r_{2}}\cdot \frac{r_{2} }{R_{0}}=-\frac{r_{2}}{\mu +\alpha +r_{2}}\cdot \frac{R_{2}}{R_{0}},\\ \varphi _{\mu }^{R_{0}}= & \frac{\partial R_{0}}{\partial \mu }\cdot \frac{\mu }{R_{0}}=-\left( \frac{\mu }{\epsilon +\mu }+1+\frac{\mu }{\mu +\alpha +r_{1} }\right) \frac{R_{1}}{R_{0}}-\left( \frac{\mu }{\epsilon +\mu }+1+\frac{\mu }{\mu +\alpha +r_{2} }\right) \frac{R_{2}}{R_{0}},\\ \varphi _{p }^{R_{0}}= & \frac{\partial R_{0}}{\partial p}\cdot \frac{p}{R_{0}}=\frac{R_{1}}{R_{0}},\quad \varphi _{q }^{R_{0}}=\frac{\partial R_{0}}{\partial q}\cdot \frac{q}{R_{0}}=\frac{R_{2}}{R_{0}},\quad \varphi _{\epsilon }^{R_{0}}=\frac{\partial R_{0}}{\partial \epsilon }\cdot \frac{\epsilon }{R_{0}}=\frac{\mu }{\epsilon +\mu }. \end{aligned}$$

**Fig. 2:**
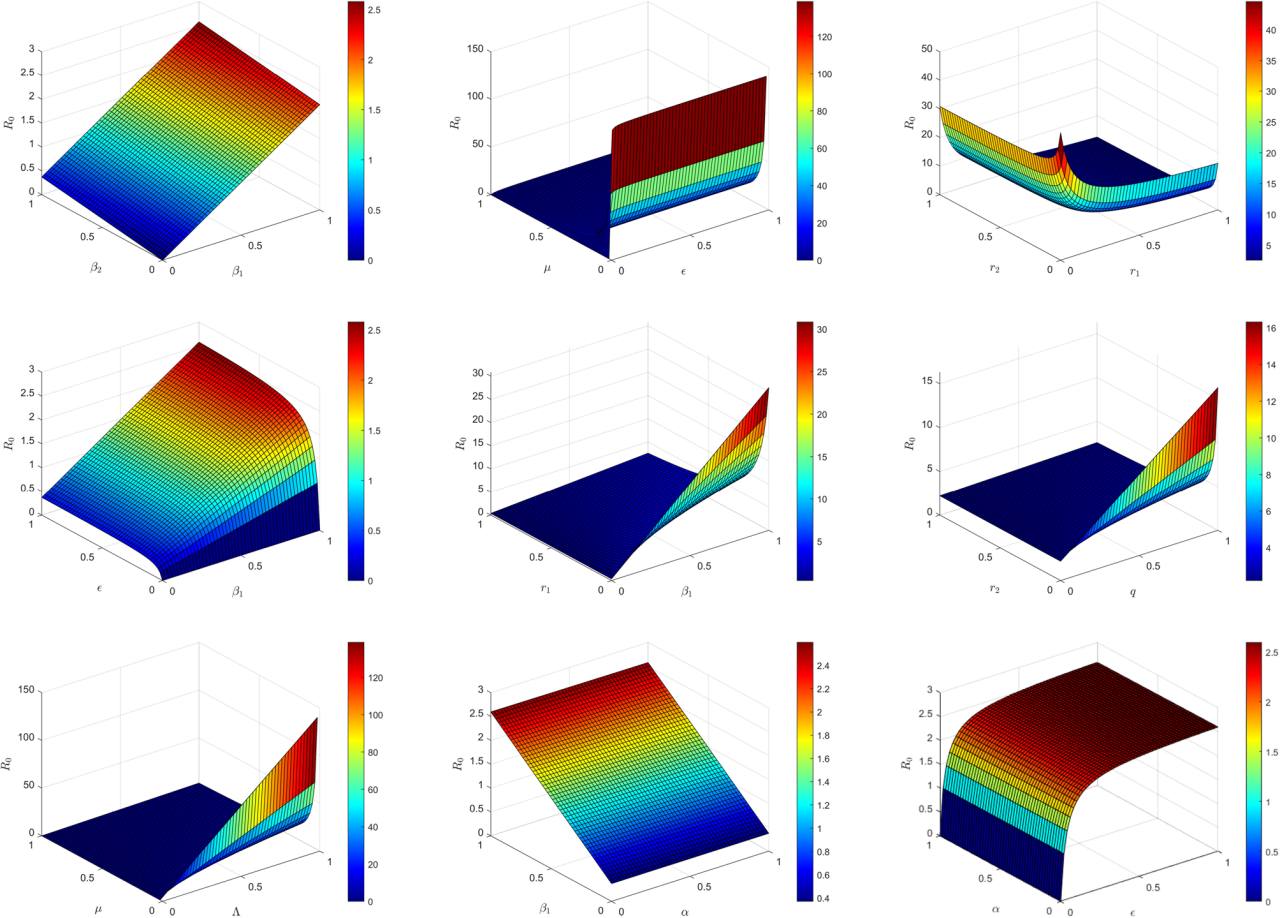
Effect of sensitive parameters.

Figure 2, it can be observed that the basic reproduction number $R_0$ has a significant positive correlation with the incidence rate parameters $\beta _1$ and $\beta _2$, indicating that a higher incidence rate is crucial for disease transmission. Furthermore, the rate of diagnosed individuals $\epsilon$ showed a positive correlation with $R_0$, while the recovery rates $r_1$ and $r_2$ exhibited a negative correlation with $R_0$, suggesting that accelerating recovery rates is a essential for controlling disease spread. The $\Lambda$ had a positive impact on $R_0$, whereas the natural mortality rate $\mu$ had a negative impact on $R_0$. Moreover, a higher disease-related mortality rate $\alpha$ significantly reduced $R_0$, indicting that while deadly diseases are dangerous, their spread may be inherently limited. Overall, the trends in the figure are highly consistent with sensitivity analysis results, emphasizing the importance of targeted interventions focusing on the incidence rate, recovery rate, and latent period for controlling disease transmission.

In summary, the transmission rates $\beta _1$, $\beta _2$, and the latent period conversion rate $\epsilon$ are the most influential parameters for $R_0$, determining the speed and severity of disease spread. Recovery rates $r_1$ and $r_2$ have a secondary influence, but they can be improved through medical interventions. The natural death rate $\mu$ and disease mortality rate $\alpha$ have less impact and are not the main intervention targets. The model’s stability is highly sensitive to $\beta$ and $\epsilon$, indicating that small changes in these parameters can significantly alter the disease transmission pattern. Therefore, public health policies should prioritize controlling and improving recovery rates to lower $R_0$ and stabilize the epidemic.

## Numerical simulations

Simulations used MATLAB R2023a on an Intel i7-11800H, with 2$\times$ longer runtime for fractional cases.

In order to keep the dimensions of the model the same, we have

10 $$\begin{aligned} \left\{ \begin{aligned}&_{0}^{C} D_{t}^{\gamma } S(t)=\Lambda ^{\gamma } -S(\beta _{1}^{\gamma } I_{1} +\beta _{2}^{\gamma } I_{2})-\mu ^{\gamma } S,\\ &_{0}^{C} D_{t}^{\gamma } E(t)= S(\beta _{1}^{\gamma } I_{1}+\beta _{2} ^{\gamma } I_{2} )-(\epsilon ^{\gamma } +\mu ^{\gamma } )E,\\&_{0}^{C} D_{t}^{\gamma } I_{1}(t)=p \epsilon ^{\gamma } E-(\mu ^{\gamma } +\alpha ^{\gamma } +r_{1}^{\gamma } )I_{1},\\&_{0}^{C} D_{t}^{\gamma } I_{2}(t)=q \epsilon ^{\gamma } E-(\mu ^{\gamma } +\alpha ^{\gamma } +r_{2}^{\gamma } )I_{2},\\&_{0}^{C} D_{t}^{\gamma } R(t)=r_{1}^{\gamma } I_{1}+ r_{2}^{\gamma } I_{2}-\mu ^{\gamma }R. \end{aligned}\right. \end{aligned}$$

System (10) consistently admits a disease-free equilibrium, denoted as $P_0 (S_0, 0, 0, 0, 0)$, where $S_0 = \frac{\Lambda ^\gamma }{\mu ^\gamma }$. The basic reproduction number corresponding to system (10) is given as follows

$$\begin{aligned} R_{0} =\frac{S_{0}\beta _{1}^{\gamma } p \epsilon ^{\gamma } }{(\epsilon ^{\gamma } +\mu ^{\gamma } )(\mu ^{\gamma } +\alpha ^{\gamma } +r_{1}^{\gamma } )} + \frac{S_{0}\beta _{2}^{\gamma } q \epsilon ^{\gamma } }{(\epsilon ^{\gamma } +\mu ^{\gamma } )(\mu ^{\gamma } +\alpha ^{\gamma } +r_{2}^{\gamma } )}. \end{aligned}$$

To optimize the analysis, we utilize the parameter settings in Table 1. Numerical computations provide the disease-free equilibrium values for different $\gamma$ levels, which are systematically listed in Table 3.

**Table 1:** The values of various parameters in system (10).

Parameters	The first set of values	The second set of values	References
$\beta _{1}$	$2.65\times 10^{-8}$	$2.65\times 10^{-11}$	^14,39^
$\beta _{2}$	$2.65\times 10^{-8}$	$2.65\times 10^{-11}$	^14,39^
$\Lambda$	100,000	100,000	^14^
$\mu$	$7.14\times 10^{-3}$	$7.14\times 10^{-3}$	^40^
$\epsilon$	0.2	0.2	^39^
*p*	0.6834	0.6834	^39^
*q*	0.3166	0.3166	^39^
$\alpha$	0.0009	0.0009	^39^
$r_{1}$	0.1029	0.1029	^39^
$r_{2}$	0.2978	0.2978	^39^
*S*(0)	11081000	11081000	^39^
*E*(0)	600	600	^39^
$I_{1}(0)$	410	410	^39^
$I_{2}(0)$	30	30	^39^
*R*(0)	2	2	^39^

Based on the information in the Table 2, we can plot the relationship between the order $\gamma$ and the basic reproduction number $R_{0}$.

**Fig. 3:**
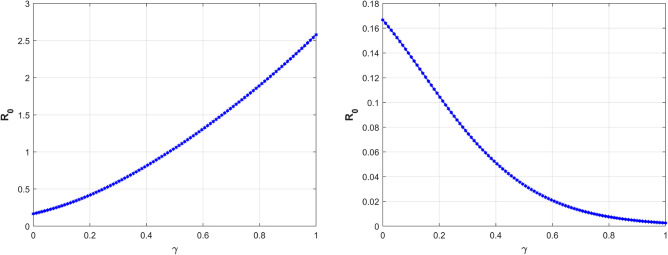
Plot of $R_{0}$ versus $\gamma$ for the first and the second set of values.

Figure 3 on the left represents the relationship between $R_0$ and $\gamma$ under the first set of parameter values, while the graph on the right illustrates the same relationship under the second set of values. From the left graph, it can be observed that for $\gamma = 0.8, 0.85, 0.9, 0.95, 1$, the basic reproduction number $R_0 > 1$. According to Theorem 4.2, this implies that the endemic equilibrium $P^*$ is globally asymptotically stable. Through further calculations, we can determine the specific endemic equilibrium and its stability for different $\gamma$ values in the first set of parameters. Similarly, for the second set of values, the right graph shows that $R_0 \le 1$ for all $\gamma$, indicating that the disease-free equilibrium $P_0$ is stable across different $\gamma$ values.

**Table 2:** Endemic equilibrium point for different $\gamma$ in the first set of values.

$\gamma$	$R_{0}$	Equilibrium point $P^{*}$	Stability
0.8	1.895960	$P^{*} = (274924, 16012, 16322, 3477, 206732)$	$P^{*}$ is GAS
0.85	2.056275	$P^{*} = (577132, 33883, 36328, 7289, 524608)$	$P^{*}$ is GAS
0.9	2.223072	$P^{*} = (1215397, 70543, 79370, 15005, 1306965)$	$P^{*}$ is GAS
0.95	2.396921	$P^{*} = (2566451, 145077, 170955, 30464, 3210473)$	$P^{*}$ is GAS
1.0	2.578462	$P^{*} = ( 5431767, 295535, 364105, 61187, 7799401)$	$P^{*}$ is GAS

**Table 3:** Disease-free equilibrium point for different $\gamma$ in the second set of values.

$\gamma$	$R_{0}$	Equilibrium point $P_0$	Stability
0.8	0.007548	$P_0 = (521244, 0, 0, 0, 0)$	$P_0$ is GAS
0.85	0.005795	$P_0 = (1186743, 0, 0, 0, 0)$	$P_0$ is GAS
0.9	0.004436	$P_0 = (2701915, 0, 0, 0, 0)$	$P_0$ is GAS
0.95	0.003386	$P_0 = (6151580, 0, 0, 0, 0)$	$P_0$ is GAS
1.0	0.002578	$P_0 = (14005602, 0, 0, 0, 0)$	$P_0$ is GAS

In Tables 2 and 3, we calculated the endemic equilibrium point and disease-free equilibrium point, further verifying the correctness of the theoretical analysis.

Taking initial value are $S(0)=11081000$, $E(0)=600$, $I_{1}(0)=410$, $I_{2}(0)=30$, $R(0)=2$, we can get a plot of *S*(*t*), *E*(*t*), $I_{1}(t)$, $I_{2}(t)$, and *R*(*t*) over time in the first set of values.

**Fig. 4:**
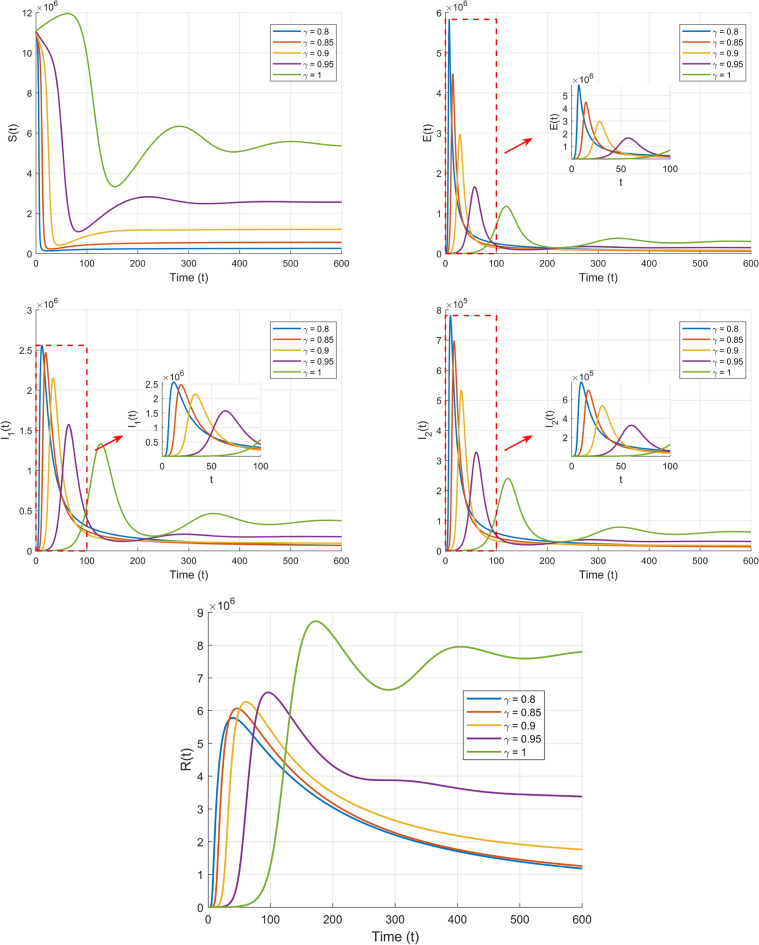
Plot of $S(t),E(t),I_{1}(t),I_{2}(t),R(t)$ in terms of *t*.

From Fig. 4, it can be observed that the fractional-order parameter $\gamma$ not only influences the dynamic behavior of the disease transmission model but also reflects the memory and nonlocal properties of fractional derivatives. Lower values of $\gamma$ (such as $\gamma = 0.8$ and $\gamma = 0.85$) enhance the system’s rapid decay characteristics, causing the number of infected individuals to decrease quickly, which indicates shorter memory effects. In contrast, higher values of $\gamma$ (such as $\gamma = 0.95$ and $\gamma = 1$) strengthen the system’s nonlocal characteristics, making the disease transmission and recovery processes slower and more prolonged. Fractional derivatives, by incorporating weighted memory of historical states, regulate the rate and scale of disease transmission, highlighting their unique advantage in describing complex dynamic systems and providing theoretical support for more precise modeling and control of disease spread.

Taking initial value are $S(0)=11081000$, $E(0)=600$, $I_{1}(0)=410$, $I_{2}(0)=30$, $R(0)=2$ we can get a plot of *S*(*t*), *E*(*t*),$I_{1}(t)$,$I_{2}(t)$, *R*(*t*) over time in the second set of values.

“*Fractional models required 1.5C2*$\times$ *more computation time than integer-order counterparts. Adaptive step sizing reduced this gap by 30% without sacrificing accuracy.*”

**Fig. 5:**
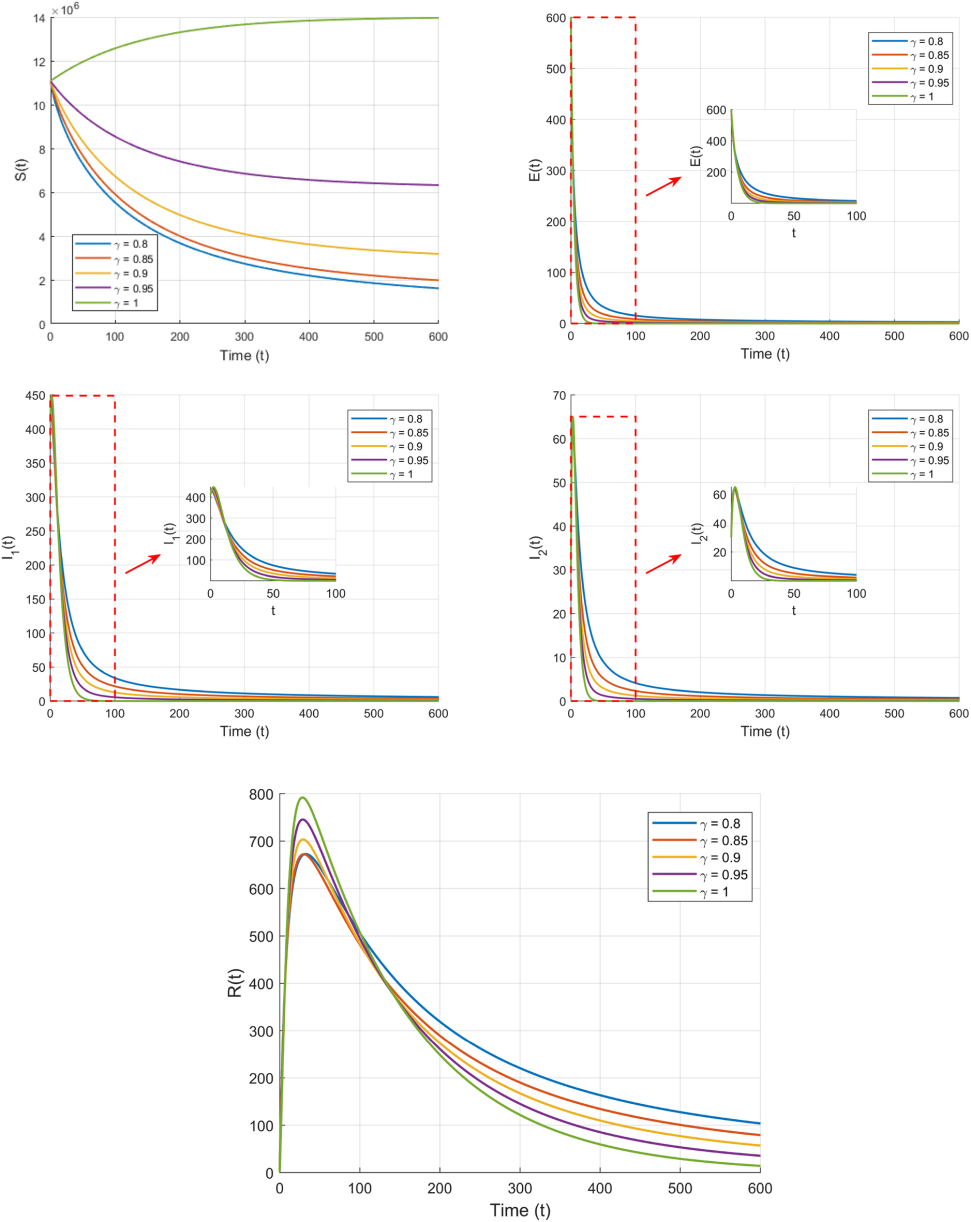
Plot of $S(t),E(t),I_{1}(t),I_{2}(t),R(t)$ in terms of *t*.

From Fig. 5, it can be observed that different fractional order parameters $\gamma$ have a significant impact on the dynamic behavior of the system. From the $E$, $I_1$, $I_2$, and $R$ plots, it is evident that the larger the order $\gamma$, the faster the changes occur, reflecting the memory and hereditary properties of fractional calculus. Lower $\gamma$ values (e.g., $\gamma = 0.8$) prolong convergence due to stronger memory effects.

Through Figs. 4 and 5, we observed that when $R_0 > 1$, the number of infected individuals stabilizes at a positive equilibrium point, while when $R_0 <1$, the number of infected individuals eventually converges to zero, which fully matches the theoretical analysis. In the figures, we respectively show the time evolution curves of susceptible S, exposed E, infected individuals (both symptomatic and asymptomatic), and recovered R under the first and second sets of parameter settings with different fractional order values.

Through the above simulation, we observe that the fractional order parameter $\gamma$ significantly influences the dynamic behavior of system (10). From the plots of $S(t)$, $E(t)$, $I_1(t)$, $I_2(t)$, and $R(t)$ with respect to $t,$ under conditions where $R_0 < 1$ or $R_0 > 1$, it is clear that the rate of change in the curves for $\gamma < 1$ differs notably from those for $\gamma = 1$. This reflects the memory and hereditary characteristics intrinsic to fractional order systems. By tuning the fractional order parameter $\gamma$, the speed and duration of disease transmission can be effectively regulated, offering a more refined and accurate modeling framework for designing disease prevention and control strategies. *Memory effects imply that control measures (e.g., lockdowns) require earlier implementation to counteract historical transmission inertia.*

## Conclusions

Many scholars have studied the dynamics of multi-disease coexistence^41^, the impact of socio-economic factors on disease transmission^42^, and virus transmission modeling incorporating deep learning and intervention measures^43^. Fantaye et al.^44^ applied the ABC fractional derivative to study the transmission of coffee berry disease (CBD). These studies provide valuable references for the expansion and optimization of our model, particularly in characterizing complex disease transmission processes, optimizing model parameters, and improving predictive accuracy.

Although this paper proposes and analyzes an SEIR model with generalized infection rate based on Caputo fractional derivatives, enriching the theoretical framework of the classical model, there are still certain limitations. Firstly, although the generalized infection rate function is more general than the classical model, we assume that the function satisfies the separable variable condition, which limits the model’s applicability to more complex infection rate functions. Secondly, this paper assumes the order of the fractional derivative to be constant, while in real epidemic dynamics, the memory effect of the system may vary over time, so it would be more reasonable to use variable-order fractional derivatives. Future research will consider more general *h*(*S*, *I*) forms and the application of variable-order fractional derivatives. For future research directions, the fractional-order model could dynamically capture the memory effects at different stages of the epidemic by introducing variable-order fractional derivatives, or better adapt to complex transmission environments by considering time-varying parameters and stochastic perturbations. Additionally, the model could be extended to a spatial fractional partial differential model to account for the impact of geographic heterogeneity, population movement, and other factors on epidemic transmission.

## Data Availability

Data sharing not applicable to this article as no datasets were generated or analyzed during the current study.
